# Reply to: Comments on “Nomograms based on inflammatory biomarkers for predicting tumor grade and micro-vascular invasion in stage I/II hepatocellular carcinoma”

**DOI:** 10.1042/BSR20193401

**Published:** 2019-12-04

**Authors:** Peng Li, Wei Huang, Feng Wang, Ye-Fang Ke, Lin Gao, Ke-Qing Shi, Meng-Tao Zhou, Bi-Cheng Chen

**Affiliations:** 1 Department of Pathology, The First Affiliated Hospital of Wenzhou Medical University, Wenzhou, China; 2 Key Laboratory of Diagnosis and Treatment of Severe Hepato-Pancreatic Diseases of Zhejiang Province, The First Affiliated Hospital of Wenzhou Medical University, Wenzhou, China; 3 Precision Medical Center Laboratory, The First Affiliated Hospital of Wenzhou Medical University, Wenzhou, China

**Keywords:** hepatocellular carcinoma, inflammatory biomarkers, micro-vascular invasion, nomogram, tumor grade

## Abstract

We appreciate to receive commentary from Dr Guangtong Deng and Dr Liang Xiao to our article, “Nomograms based on inflammatory biomarkers for predicting tumor grade and micro-vascular invasion in stage I/II hepatocellular carcinoma”. First, neutrophil-to-lymphocyte ratio (NLR) and derived NLR (dNLR) are two different parameters. Some studies show that NLR is inconsistent with dNRL in prognostic value through multivariate Cox regression, therefore, it is reasonable that both NLR and dNLR entered into multivariate analysis simultaneously. Second, it is common that articles of predictive nomograms turned continuous variables into categorical variables. The reason is that the categorization of patient clinical variables is beneficial to doctors to make decisions based on the risk level of individual patients in clinical. At last, multicenter validation is quite difficult and we have listed the shortcomings in the limitations of our article. Further validation will need the joint efforts by other institutions.

We thank Dr Guangtong Deng and Dr Liang Xiao for their valuable comments and suggestions on “Nomograms based on inflammatory biomarkers for predicting tumor grade and micro-vascular invasion in stage I/II hepatocellular carcinoma [1]”.

First, neutrophil-to-lymphocyte ratio (NLR) is equal to neutrophil divided by lymphocyte. However, derived neutrophil-to-lymphocyte ratio (dNLR) is that neutrophil count divided by the result of white cell count minus neutrophil count [2]. NLR and dNLR are two different parameters. Furthermore, some studies show that NLR is inconsistent with dNRL in prognostic value through multivariate Cox regression [2–8]. Therefore, it is reasonable that both NLR and dNLR entered into multivariate analysis simultaneously.

Second, 86% of epidemiological investigations classified continuity factors to categorical variables [9]. The advantages of categorization are listed as follows: (1) The results may be more readily understood by non-statisticians. (2) Categorization may remove the need for any parametric assumptions regarding the shape [9]. In clinical, categorization is easily obtained and the categorization of patient clinical variables is conducive for doctors to make decisions based on the risk level of individual patients [10]. In addition, many excellent articles of predictive nomograms also turned continuous variables into categorical variables [11,12]. We have mentioned the setting of the cut-off values in the method of our article – ‘Receiver operating characteristic (ROC) curve analysis was used to calculate the optimal cutoff values that were determined by maximizing the Youden index (i.e., sensitivity+specificity-1). Accuracy of the optimal cutoff value was assessed by the sensitivity, specificity, predictive values, and likelihood ratios’.

Last, the purpose of our article is to develop nomograms predicting tumor grade and MVI based on inflammatory biomarkers with high accuracy. Due to experimental resource limitations, the step of an independent dataset validation is not included. As most published articles, we have listed the shortcomings, lacking of validation by other research institutes, in the limitations of our article.

## Abbreviations

dNLR: derived neutrophil-to-lymphocyte ratio
MVI: micro-vascular invision
NLR: neutrophil-to-lymphocyte ratio
ROC: receiver operating characteristic
